# *Vibrio cholerae* Persisted in Microcosm for 700 Days Inhibits Motility but Promotes Biofilm Formation in Nutrient-Poor Lake Water Microcosms

**DOI:** 10.1371/journal.pone.0092883

**Published:** 2014-03-25

**Authors:** Mohammad Jubair, Kalina R. Atanasova, Mustafizur Rahman, Karl E. Klose, Mahmuda Yasmin, Özlem Yilmaz, J. Glenn Morris, Afsar Ali

**Affiliations:** 1 Department of Environmental and Global Health, School of Public Health and Health Professions, University of Florida at Gainesville, Gainesville, Florida, United States of America; 2 Department of Periodontology, University of Florida at Gainesville, Gainesville, Florida, United States of America; 3 Department of Biology, The University of Texas at San Antonio, Texas, United States of America; 4 Department of Microbiology, University of Dhaka, Dhaka, Bangladesh; 5 Emerging Pathogens Institute, University of Florida at Gainesville, Gainesville, Florida, United States of America; State Key Laboratory of Pathogen and Biosecurity, Beijing Institute of Microbiology and Epidemiology, China

## Abstract

Toxigenic *Vibrio cholerae*, ubiquitous in aquatic environments, is responsible for cholera; humans can become infected after consuming food and/or water contaminated with the bacterium. The underlying basis of persistence of *V. cholerae* in the aquatic environment remains poorly understood despite decades of research. We recently described a “persister” phenotype of *V. cholerae* that survived in nutrient-poor “filter sterilized” lake water (FSLW) in excess of 700-days. Previous reports suggest that microorganisms can assume a growth advantage in stationary phase (GASP) phenotype in response to long-term survival during stationary phase of growth. Here we report a *V. cholerae* GASP phenotype (GASP-700D) that appeared to result from 700 day-old persister cells stored in glycerol broth at −80°C. The GASP-700D, compared to its wild-type N16961, was defective in motility, produced increased biofilm that was independent of *vps* (p<0.005) and resistant to oxidative stress when grown specifically in FSLW (p<0.005). We propose that *V. cholerae* GASP-700D represents cell populations that may better fit and adapt to stressful survival conditions while serving as a critical link in the cycle of cholera transmission.

## Introduction

Cholera is a major public health threat worldwide, particularly in countries where safe drinking water, adequate sanitation and hygiene are suboptimal [1]. Cholera toxin (CT)-producing *V. cholerae* strains, generally in serogroups O1 and O139, are the cause of epidemic cholera. *V. cholerae* has two life styles, including transient passage through the human intestine where it causes profuse diarrhea (i.e. cholera), and a second existence in aquatic environments, including fresh, estuarine and marine environments [1], [2], [3]. In aquatic reservoirs, the microorganism can survive either in planktonic (free-living) form or in biofilms [2], [3]. Available data suggest that the bacteria survive between epidemics in these aquatic reservoirs, with environmental triggers causing seasonal increases in counts, followed by “spill-over” into human populations [1]. The genetic and physiologic basis of persistence of *V. cholerae* in the environments, particularly during inter-epidemic period, is poorly understood.

In this context, it has been suggested that *V. cholerae* can enter into a viable but non-culturable state (VBNC) in response to nutrient starvation and/or cold temperature [4], [5]; however, the resuscitation of VBNC, under laboratory conditions, is inconsistent, raising questions about the role of the VBNC state in cholera epidemiology [6], [7]. *V. cholerae* can also switch from a smooth colony type to a “rugose” (wrinkled) variant characterized by copious production of exopolysaccharide conferring resistance to chlorine, osmotic and oxidative stresses [8], [9], [10]. However, the role(s) of rugose variant of *V. cholerae* in epidemic cholera is limited because not all *V. cholerae* strains are capable of switching to rugose variant even in a medium promoting high-frequency rugose production [9].

Amid this conundrum, we recently reported that a subset of culturable *V. cholerae* assume what we have termed a “persister” phenotype in a “filter sterilized” lake water (FSLW) microcosm model [11]. In that study we found that only 13% of the microcosms yielded cells that persisted in excess of 700 days while 87% of the microcosms resulted in the death of cells by 120 days. Furthermore, we observed that persisting cells in 700-day old microcosms expressed a small colony phenotype associated with very small rod shaped cells with peritrichous flagella and a high degree of cell aggregation. In contrast, cells persisting in microcosms for 24 h exhibited normal colony phenotype with heterogeneous mixtures of cells with predominantly long helical cells with bipolar flagella [11]. A “growth advantage in stationary phase” (GASP) phenotype describes microorganisms that survive long-term in a stationary growth phase under stressful conditions [12], [13], [14]. For further analysis of 700 day-old cells, we subcultured the cells from microcosms onto L-agar and subsequently stored them in glycerol broth at −80°C. As we were not certain if 700 day-old persister cells of microcosm origin will retain their genetic and phenotypic traits unchanged upon storage in glycerol broth, for our convenience, we refer this glycerol-stored cells to GASP-700D phenotype; in contrast, wild-type *V. cholerae* N16961S strain grown overnight statically in FSLW at room temperature will be henceforth termed as N16961S-24 (Table1).

Persister cells in other human pathogens exhibited biofilm formation conferring resistance to environmental stresses [15], [16], [17], [18]. In *V. cholerae* the positive association of polar flagellum to biofilm formation has been demonstrated [19]. To better understand the GASP-700D phenotype of *V. cholerae* and to compare the differences, if any, between N16961S-24 and GASP-700D, we investigated the role(s) of novel flagella elicited by N16961S-24 and GASP-700D, respectively [11], in motility and biofilm formation. Here, we provide evidence that GASP-700D showed no motility in soft agar; produced biofilm only in nutrient-poor FSLW; and conferred resistance to oxidative stress when compared to N16961S-24.

## Materials and Methods

### Bacterial Strains and Growth Conditions

Bacterial strains, including *V. cholerae* wild-type strain N16961S and its isogenic mutants (obtained either natural selection and/or created by defined genetic mutations) used in this study are listed in Table 1. As reported earlier, we generated *V. cholerae* N16961 persister cells (in excess of 700 days) in “filter sterilized” lake water microcosm model. Briefly, aliquots (500 ml) of lake water were sterilized using Nalgene 0.22 μm membrane filter units (Nalgene), and the microcosms were prepared as follows: 50 ml of “filter sterilized” lake water (FSLW) was transferred into a sterile 250 ml Erlenmeyer flask; for inoculum preparation a single colony of *V. cholerae* N16961strain, obtained from L-agar grown overnight at 37°C, was inoculated into 3 ml of L-broth. The culture was incubated overnight at 37°C with a shaking speed of 250 rpm, spun down and the resulting pellet was washed 2X in saline (0.85% NaCl), reconstituted in 3 ml saline, appropriately diluted, and 50 μl of diluted culture was inoculated into the microcosm flasks containing 50 ml FSLW. As confirmed by plate counts, initial *V. cholerae* concentrations in the microcosms ranged from 10^4^ to 10^6^ cfu/ml. The culturable cells from microcosm were determined at intervals using standard plate counts. The 700 day-old cells were subcultured on L-agar and stored in glycerol broth at −80°C. While we cannot be certain that this is true for all GASP-700D cells, we observed that GASP-700D exhibited small colony phenotypes on L-agar for at least four consecutive days of subculture both at room temperature and at 37°C. However, when the cells were inoculated into L-broth and incubated overnight at 37°C with a shaking speed of 250 rpm, a mixture of small and large colonies were observed on L-agar upon plating. All the strains used in this study were subcultured from glycerol stock at −80°C onto L-agar and incubated overnight at 37°C before being used for specific experiments. As needed, antibiotic was added to the bacterial cultures as follows: ampicillin (100 μg/ml) and polymyxin B (50 U/ml).

**Table 1:** Bacterial strains and plasmids used in this study.

Strain orPlasmid	Description	Reference
*V. cholerae*strains		
N16961S	A wild-type, smooth, O1 El Tor strain isolated in Bangladesh in 1971	[9]
N16961S-24	A growth of N16961S in nutrient-poor “filter sterilized” lake water incubatedovernight statically at room temperature	This study
N16961R	A rugose variant of N16961S strain	[24]
N16961R-24	A growth of N16961R in nutrient-poor “filter sterilized” lake waterincubated overnight statically at room temperature	This study
GASP-700D	700 days-old N16961S culture persisting in nutrient-poor FSLW was grown onL-agar and subsequently stored in FSLW supplemented with30% glycerol at −80°C	[11]
AA212	A Δ*flaA* null mutation in the background of N16961S strain	This study
AA215	A SΔ*vpsR* (420 bp internal in-frame deletion) created in the N16961S strain	This study
AA216	A RΔ*vpsR* (420 bp internal in-frame deletion) created in the N16961R	This study
AA217	A GASP-700DΔ*vpsR* (420 bp internal in-frame deletion) created in the background ofGASP-700D strain	This study
AA218	A SΔ*vpsA* (VC_0917) in-frame null mutation was created in N16961S strain	This study
AA219	A RΔ*vpsA* (VC_0917) in-frame null mutation was created in N16961R strain	This study
AA220	A GASP-700DΔ*vpsA* (VC_0917) in-frame null mutation was created in the background ofGASP-700D strain	This study
*E. coli* strains		
DH5α	*recA* Δ*lac*U169 φ80d *lacZ*ΔM15	Gibco, BRL
S17-1 λ *pir*	*Pro hsdR hsdM* ^+^ Tmp^r^ Str^r^	[41]
Plasmids		
pWSK29	Low-copy-number vector, Amp^r^, *ori* pSC101	[23]
pCVD442	Suicide vector, *ori* R6K, Amp^r^, *sacB*	[42]
pKEK93	Δ*flaA*::Cm in pCVD442	[20]
pAA69	A 560-bp PCR fragment (*SacII/XbaI)* containing the 5′-end of *vpsR* gene of N16961cloned into similarly digested pWSK29, Amp^r^	This study
pAA72	A 540-bp PCR fragment (*SacII-SpeI)* fragment upstream of *vpsA* gene of N16961cloned into similarly digested pWSK29, Amp^r^	This study
pAA73	A 520-bp PCR fragment (*XbaI-BamH1)* was cloned into similarly digested pAA69, resultingin a plasmid (pAA73) containing 420-bp internalin-frame deletion. Amp^r^	This study
pAA74	A 360-bp PCR fragment (*SpeI-EcoR1)* downstream of *vpsA* was cloned into similarlydigested pAA72, resulting in a plasmid (pAA74). Amp^r^	This study
pAA77	A 900-bp PCR fragment (*SacI-SalI)* from pAA74 was cloned into similarlydigested pCVD442, Amp^r^	This study
pAA78	A 1080-bp PCR fragment (*SacI-SalI)* from pAA73 was cloned into similarlydigested pCVD442, Amp^r^	This study

### Genetic Manipulations

A Δ*flaA* mutant (AA212; Table 1) was created in the background of N16961S strain (Table 1) using a Δ*flaA* gene targeting vector described previously [20]. For creating in-frame mutation in *hydG*/*vpsR* [8], [21] and in a rugosity-associated gene, *vpsA* (VC0917, encoding UDP-N-acetylglucosamine 2-epimerase [*wecB*]) [22] in the back ground of N16961S, N16961R and GASP-700D (Table 1), a two-step PCR cloning strategy was used. Briefly, two PCR products flanking an internal deletion (420-bp) in *vpsR* were engineered. Each PCR product carries a restriction endonuclease site at its 5′ end; however, 3′-ends of the forward and reverse PCR products carried a common restriction site to facilitate deletion mutation. For *vpsR*, *SacII* and *XbaI* sites were introduced at 5′ and 3′ ends, respectively, of the forward PCR amplicon (560-bp) while 5′ and 3′ ends of reverse PCR product (520-bp) were introduced with *XbaI* and *BamHI* sites, respectively. Primers aa212 and aa213 (Table S1) were used to amplify forward PCR fragment using N16961S chromosomal DNA as a template with standard PCR conditions. The PCR product was purified using Qiaquick PCR purification kit (Qiagen, Valencia, CA). The purified PCR product was digested with *SacII* and *XbaI,* the digested product was purified, and the PCR product was ligated with a similarly digested vector, pWSK29, [23] resulting in a plasmid (pAA69). The plasmid was transformed into *Escherichia coli* DH5α as described previously [24]. Next, two convergent PCR primers, including aa214 and aa215 (Table S1) were used to amplify the reverse PCR product; the amplicon was purified and digested with *XbaI* and *BamHI*. The digested products were purified and ligated into a similarly digested plasmid (pAA69), resulting in a plasmid pAA73 containing a 420-bp internal deletion of *vpsR*. The plasmid was transformed into DH5α. Subsequently, plasmid pAA73 was digested with *SacI* and *SalI* to retrieve a 1080-bp fragment and the fragment was gel purified. The purified fragment was ligated into a similarly digested suicide vector, pCVD442, [25] and transformed into an *E. coli* S17 λ *pir* resulting in a plasmid pAA78 (Table 1). *E. coli* S17 λ *pir* carrying pAA78 was conjugated to *V. cholerae* N16961S, N16961R and GASP-700D. Selection of transconjugants, counter selection, and chromosomal mutation using homologous recombination of *vpsR* was performed as described previously [21], [24]. Mutants sustained an internal in-frame deletion in *vpsR* (SΔ*vpsR,* mutation in smooth background [AA215, Table 1], RΔ*vpsR*, mutation in rugose background [AA216] and GASP-700DΔ*vpsR,* mutation in GASP-700D background [AA217]) were verified by PCR and DNA sequencing as described previously [24]. A similar approach was also used for creating a null mutation in the *vpsA* gene in the background of N16961S, N16961R and GASP-700D, resulting in the mutants AA218, AA219 and AA220, respectively. Primers (aa264 and aa265, aa266 and 267) used to create null mutation in *vpsA* are listed in Table S1.

### Motility Assay

Motility of *V. cholerae* strains was determined using motility agar plates as described previously [24] with minor modifications. The experiment was performed with cells grown both in L-broth and FSLW. Briefly, N16961S, N16961R, GASP-700D and Δ*flaA* mutant were grown in L-broth and incubated overnight statically at room temperature. As for FSLW, the strains were first subcultured onto L-agar; a single colony from L-agar was grown in 3 ml L-broth and incubated overnight statically at room temperature. Subsequently, the cultures were spun down at 7,000 rpm for 5 min in a table top centrifuge; the pellet was washed 2X with FSLW and resuspended into 3 ml FSLW and the culture was incubated overnight statically at room temperature. An inoculating wire was dipped into each culture and then stabbed into the motility agar plate. The plates were incubated for 8 h and overnight at 37°C. Zones of migration of bacterial strains around the inoculating sites were measured at 8 h and after overnight incubation of the plates. If no zone was detected, a block of agar was cut around the inoculating site, homogenized in saline (0.85% NaCl), appropriately diluted in saline, and then plated on L-agar to determine if any culturable cells were survived in the inoculation site.

### Quantitative Real-time Reverse Transcription PCR (qRT-PCR)

For qRT-PCR, *V. cholerae* strains, including N16961S-24, N16961R-24 and GASP-700D (Table 1) were grown in FSLW overnight statically at room temperature. Total RNA was extracted and purified from each culture using the RNeasy kit (Qiagen, Valencia, CA); the contaminating DNA in the preparation was eliminated on-column by DNase digestion. Total RNA (10 ng) was converted to cDNA, and the RT-PCR assay were performed using iScript one-step RT-PCR kit with SYBR green (Bio-Rad, Hercules, CA) and CFX96 Real-Time PCR System (Bio-Rad, Hercules, CA) following manufacturer’s instructions. Primers used in this study are listed in Table S1. For each sample, the mean cycle threshold of the test transcript was normalized to that of *toxR* (*toxR* was equally expressed both in L-broth and in FSLW) and presented relative to *V. cholerae* N16961S-24 strain that has arbitrarily been taken as 1 (Figure S1). Values above 1 or less than 1of a selected gene indicate that the transcript was present in higher or lower numbers, respectively, than that of control strain. Data are based on three independent experiments. Previous report using qPCR demonstrated that *V. cholerae* expressed *phoB* and Pst-system genes while repressed *tcp* genes when grown in “filter sterilized” pond water microcosms compared to its growth in nutrient-rich L-broth [7]. To validate our qRT-PCR data, we compared the differential gene expression by growing *V. cholerae* N16961S strain in nutrient-rich L-broth incubated overnight statically, and in nutrient-deficient FSLW under identical growth conditions. Expression of transcripts was determined as described above except that the threshold of transcript was presented relative to *V. cholerae* N16961S strain grown in L-broth.

### Biofilm Assays

Quantitative assessment of biofilm produced by *V. cholerae* strains grown both in L-broth and in FSLW was measured as described previously [19] with modifications. Twenty-four well polystyrene plastic plates (Corning Incorporated, Corning, NY) were used as the surface for bacterial attachment. For assessment of biofilm produced in L-broth, *V. cholerae* strains, including N16961S, SΔ*vpsR*, SΔ*vpsA*, N16961R, RΔ*vpsR*, RΔ*vpsA*, GASP-700D, GASP-700D Δ*vpsR* and GASP-700DΔ*vpsA* (Table 1) were examined. Biofilm assay was performed as described previously [19]. For measurement of biofilm produced in FSLW, *V. cholerae* strains, including N16961S-24, SΔ*vpsR*, SΔ*vpsA*, N16961R-24, RΔ*vpsR*, RΔ*vpsA*, GASP-700D, GASP-700DΔ*vpsR* and GASP-700DΔ*vpsA* (Table 1) were investigated. Briefly, a single colony of each strain grown overnight on L-agar was inoculated into 3 ml L-broth and the cultures were incubated overnight with shaking (250 rpm) at 37°C. The culture was spun down and the pellete was washed 2X with FSLW and subsequently reconstituted into 3 ml FSLW. Fifty μl culture was then mixed to 450 μl fresh FSLW (ca. 10^8^ cfu/ml) in a well of plastic plate; the culture was incubated overnight statically at room temperature. Following overnight incubation the cultures were discarded, and the wells were rinsed vigorously with distilled water to remove non-adherent cells, filled with 600 μl of a 0.1% crystal violet solution (Sigma, St. Louis, MO), allowed to incubate for 30 min at room temperature, and the wells were again rinsed vigorously with water. Quantitative biofilm formation was determined by measuring the optical density at 570 nm (OD 570) of a solution produced by extracting cell-associated dye with 600 μl of dimethyl sulfoxide (DMSO) (Sigma, St. Louis, MO).

### Confocal Microscopy

To perform confocal microscopic analysis on possible biofilm formation by *Vibrio cholerae* strains, including N16961S-24, SΔ*vpsR*, SΔ*vpsA*, N16961R-24, RΔ*vpsR*, RΔ*vpsA*, GASP-700D, GASP-700DΔ*vpsR* and GASP-700DΔ*vpsA* (Table 1) were grown (ca. 10^8^ cfu/ml) in 4-well cell culture plates (Thermo Scientific Nunc, Pittsburgh, PA) containing 500 μl FSLW. To provide bacterial attachment platform, a 12 mm round glass cover slip (Warner Instruments, Hamden, CT) was dipped into each culture well, and the cultures were incubated overnight statically at room temperature. Next day, the cover slips were washed three times with Dulbecco’s PBS (DPBS) (HyClone Laboratories, Logan, UT), and fixed in 10% neutral buffered formalin solution (Sigma-Aldrich, St Louis, MO). They were washed again with DPBS and stained using 300 μl/well of 1∶1000 SYTO 9 dye (LIVE/DEAD BacLight Bacterial Viability kit, Invitrogen, Grand Island, NY). Following three consecutive DPBS washings, glass cover slips were mounted onto 75×25 mm microscopic slides (Corning Inc., Corning, NY). The cover slips were analyzed on a confocal microscope (Leica Microsystem, Buffalo Grove, IL) with an excitation and emission wavelengths of 484 and 500 nm, respectively. The biofilm thickness was measured as an average of 5 non-overlapping fields per slide with a 20X HCX PL APO lambda blue magnifying objective. Images were digitally reconstructed with z-projections of x–y sections using Leica Application Suite Advanced Fluorescence (Leica Microsystem, Buffalo Grove, IL) and DAIME softwares [26]. The volumes of biofilms were calculated as follows: the x–y areas of each z-section were measured using ImageJ (National Institute of Mental Health, Bethesda, Maryland, USA) and were multiplied by the value of the z-step to obtain the volume of the biofilm at each section. Total biofilm volumes were calculated as a sum of the separate volumes of the z-sections as described previously [27]. At least two biological replicates were used in the imaging processes.

### Transmission Electron Microscopy (TEM)

For transmission electron microscopic (TEM) analysis, *V. cholerae* strains N16961S-24, N16961R-24 and GASP-700D (Table 1) were grown in 3 ml FSLW and the cultures were incubated overnight statically at room temperature. Material from each culture was subjected to ruthenium red staining and the stained cells were examined using TEM for the presence of bacterial exopolysaccharide as described previously [9]. In brief, the cultures were fixed in a solution of 2% glutaraldehyde-50 mM lysine-500 ppm ruthenium red in 0.1 M cacodylate buffer (pH 7.2) for 1-hr at room temperature followed by an overnight incubation at 4°C. Samples were then washed twice in 0.1 M cacodylate buffer, pelleted and encapsulated in 3% low-temperature gelling agarose type VII (Sigma-Aldrich, St. Louis, MO). The following steps were processed with the aid of a Pelco BioWave Pro laboratory microwave (Ted Pella, Redding, CA, USA). Fixed cells were post-fixed with 1% buffered osmium tetroxide one minute in hood, 45 seconds at 100 W under vacuum three minutes in hood, water washed, dehydrated in a graded ethanol series 25%, 50%, 75%, 95%, 100% followed by 100% acetone, 1X each 45 sec at 180 W. Dehydrated samples were infiltrated in a graded acetone/spurr’s epoxy resin 30%, 50%, 70%, 100%, 100%, 1X each, three minutes at 220 W under vacuum followed by 10 minutes on bench top. Resin infiltrated cells were cured at 60°C for 2 days. Cured resin blocks were trimmed, thin sectioned and collected on formvar copper 100 mesh grids, post-stained with 2% aq. Uranyl acetate and Reynold’s lead citrate. Sections were examined with a Hitachi H-7000 TEM and digital images were acquired with a Veleta camera and iTEM software.

### Stress Resistance Assay

Stress resistance of GASP-700D, including both oxidative and osmotic stresses, and stress to chlorine was assessed as described earlier [9], [10]. N16961R-24 and N16961S-24 were used as positive and negative controls, respectively. Bacterial inoculum (ca. 10^8^ cfu/ml) was inoculated in 3 ml FSLW and incubated as described above. For oxidative stress, 20 mM H_2_O_2_ (final concentration) (hydrogen peroxide 3%, Ricca Chemical, Arlington, TX) was added to each culture and the resistance of each culture to H_2_O_2_ was recorded for every 5 min for 15 min. The culturable bacteria survived the stress were determined using standard plate count at each experimental time point. Similarly, for osmotic and chlorine stresses, *V. cholerae’s* culture in FSLW was exposed to 2.5 M NaCl (at final concentration) (Avantor Performance Materials, Center Valley, PA) and 3 mg free chlorine per liter [3 ppm] (sodium hypochlorite, Sigma, St Louis, MO), respectively. Resistance of each *V. cholerae* strain to osmotic and chlorine stresses was determined by measuring the culturable bacteria present (i) for every 15 min for one hour [10], and (ii) for every 5 min for 15 min [9], respectively. Percent survival of the bacteria was calculated by dividing the number of bacterial colonies counted at a given time by the number of colonies added to the culture before supplementing the culture with stress ingredient, and then multiplying the result by 100.

### Statistical Analysis

One-way ANOVA was performed in STATA v 12 (StataCorp, College Station Texas, USA) to determine the significant differences in diverse traits assessed in the study. Equal variance within groups was assessed using Barlett’s test, and a Bonferroni correction was implemented to control type I error for multiple comparisons between the wild-type and its isogenic mutants or variants. A p-value of <0.005 was considered as statistically significant.

## Results

### Comparison of Motility between N16961S-24 and GASP-700D of *V. cholerae*

**Figure 1:**
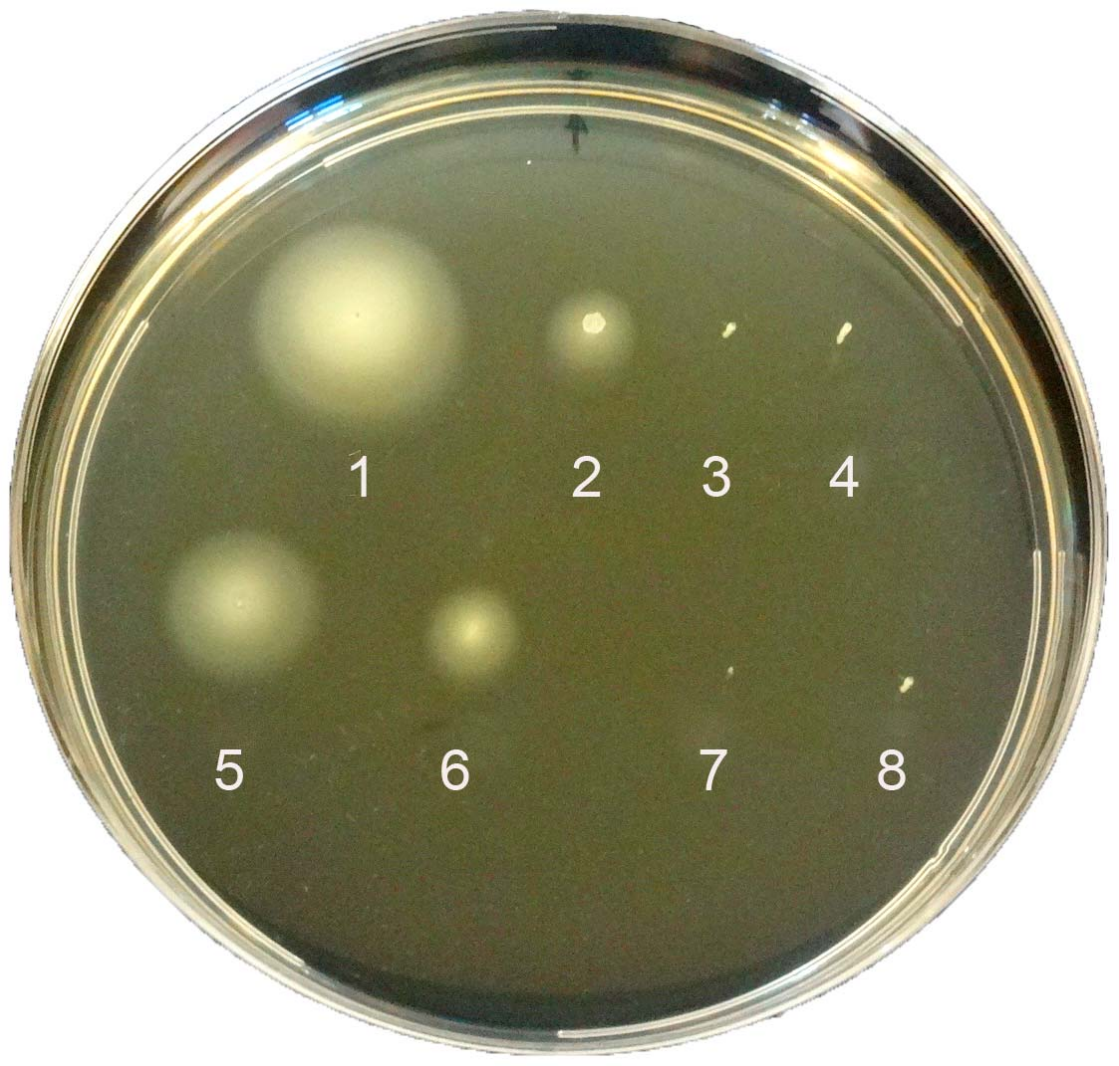
Swimming behavior of *V. cholerae* strains in motility agar. The bacterial strains were grown either in L-broth (L) or in FSLW (LW) and incubated overnight statically at room temperature before inoculating into motility agar. After inoculation, the plates were incubated at 37°C for 8 h before obtaining the images. 1, N16961S (L); 2, N16961R (L); 3, GASP-700D (L); 4, SΔ*flaA* (L); 5, N16961S (LW); 6, N16961R (LW); 7, GASP-700D (LW); 8, SΔ*flaA* (LW).

*Vibrio cholerae* carries a single polar flagellum required for its motility. Since we are the first to describe that *V. cholerae* can switch, in response to nutrient-starvation in FSLW, from a canonical single polar flagellum to bipolar and peritrichous flagella in N16961S-24 and GASP-700D, respectively [11], we were interested to investigate the role(s) of bipolar and peritrichous flagella, if any, in motility using motility agar. We also included a *ΔflaA* mutant strain that is non-motile because it lacks the major flagellin subunit [20], and a (motile) rugose variant of *V. cholerae* (N16961R). When the bacterial strains were grown in L-broth before inoculating into motility agar, both N16961S (smooth variant) and N16961R (rugose variant) were motile (Figure 1, #1 and #2), with the rugose variant exhibiting approximately 2.5-fold reduced motility, which is consistent with previous reports described by our group and others [9], [28]. To our surprise GASP-700D was non-motile (Figure 1, #3). As expected, the Δ*flaA* mutant was non-motile (Figure 1, # 4). When grown in nutrient-poor FSLW, both N16961S-24 and N16961R-24 strains demonstrated motility, with N16961S-24 exhibiting increased motility compared to the rugose variant (Figure 1, # 5 and #6) further corroborating that the rugose variant is less motile than its smooth counterpart. Interestingly, GASP-700D, in contrast to N16961S-24, did not move from the point of inoculation, even after 24 h of growth in motility agar (Figure 1, # 7). As expected, the Δ*flaA* mutant was non-motile (Figure 1, # 8). Our data suggest that unlike the bipolar flagella of N16961S-24, GASP-700D did not facilitate productive motility both in L-broth and FSLW. To ensure that GASP-700D was viable at the inoculation site, we examined a block of agar consisting of the entire inoculation site as described in methods section. We obtained ca. 1×10^6^ cfu, confirming that GASP-700D was surviving inside the agar but defective in motility.

As GASP-700D exhibited no motility in soft agar, we further investigated using qRT-PCR to determine whether flagellar genes, including *flaA* (encodes critical flagellin), *flrC* (encodes regulator of Class III flagellar genes), *motY* and *motB* (encode flagellar motor) and *flrA* (encodes master regulator of all flagellar genes) were repressed in GASP-700D relative to N16961S-24. A previous study reported that *phoB* and Pst system genes of *V. cholerae* were expressed in nutrient-poor FSLW compared to nutrient-rich L-broth, whereas *ctxA* and *tcp* genes were repressed under the same conditions [7].

**Figure 2:**
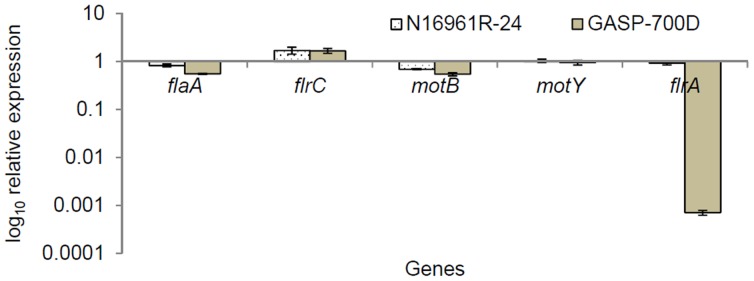
Comparative expression analysis of selected flagellar genes as measured by qRT-PCR among *V. cholerae* strains, including N16961S-24, N16961R-24 and GASP-700D. Each strain (ca. 10^8^ cfu/ml) was grown in nutrient-poor FSLW and incubated overnight statically at room temperature. Expression of each gene was normalized to that of *toxR*, and subsequently the expression of the gene was compared to that of the wild-type N16961S-24. Number one (1) represents the value of expressed gene by N16961S-24. Values above 1 or below 1 represent the positive and negative expression, respectively. Data represent the average results of at least three independent experiments are expressed as means ± standard deviation (SD). P-values are computed by comparing the differential expression of each gene with that of N1961S-24 using one-way ANOVA test. A p-value of <0.005 was considered statistically significant.

We first compared the relative expression of *phoB*, Pst-system genes, *ctxA* and *tcp* genes by N16961S-24 and GASP-700D grown in nutrient-poor FSLW to that of wild-type *V. cholerae* N16961S grown in nutrient-rich L-broth in otherwise identical growth conditions (Figure S1). The *phoB* and Pst-system genes were highly expressed, while *tcp* genes and *ctxA* were repressed, by N16961S-24 and GASP-700D grown in FSLW relative to N16961S grown in nutrient-rich L-broth, confirming the results of the previous study [7]. Additionally, expression of the flagellar genes, except *flrC*, was also down-regulated in GASP-700D, as well as in the rugose N16961R-24 variant, compared to their expression in N16961S-24 when grown in FSLW. Strikingly, *flrA*, the master flagellar regulatory gene, was 1,000-fold down regulated (p<0.005) in GASP-700D compared to N16961S-24, suggesting that flagellar synthesis is down-regulated in GASP-700D (Figure 2). Taken together, our results suggest that GASP-700D may have lost peritrichous flagella and/or some flageller gene(s) might have sustained mutation(s) in GASP-700D resulting in the defect of productive motility. Indeed, microorganisms surviving for long-time in stressful stationary growth cultures can results in the selection of mutants that express GASP phenotype [12].

### Comparative Assessment of Biofilm Formation between N16961S-24 and GASP-700D of *V. cholerae*

**Figure 3:**
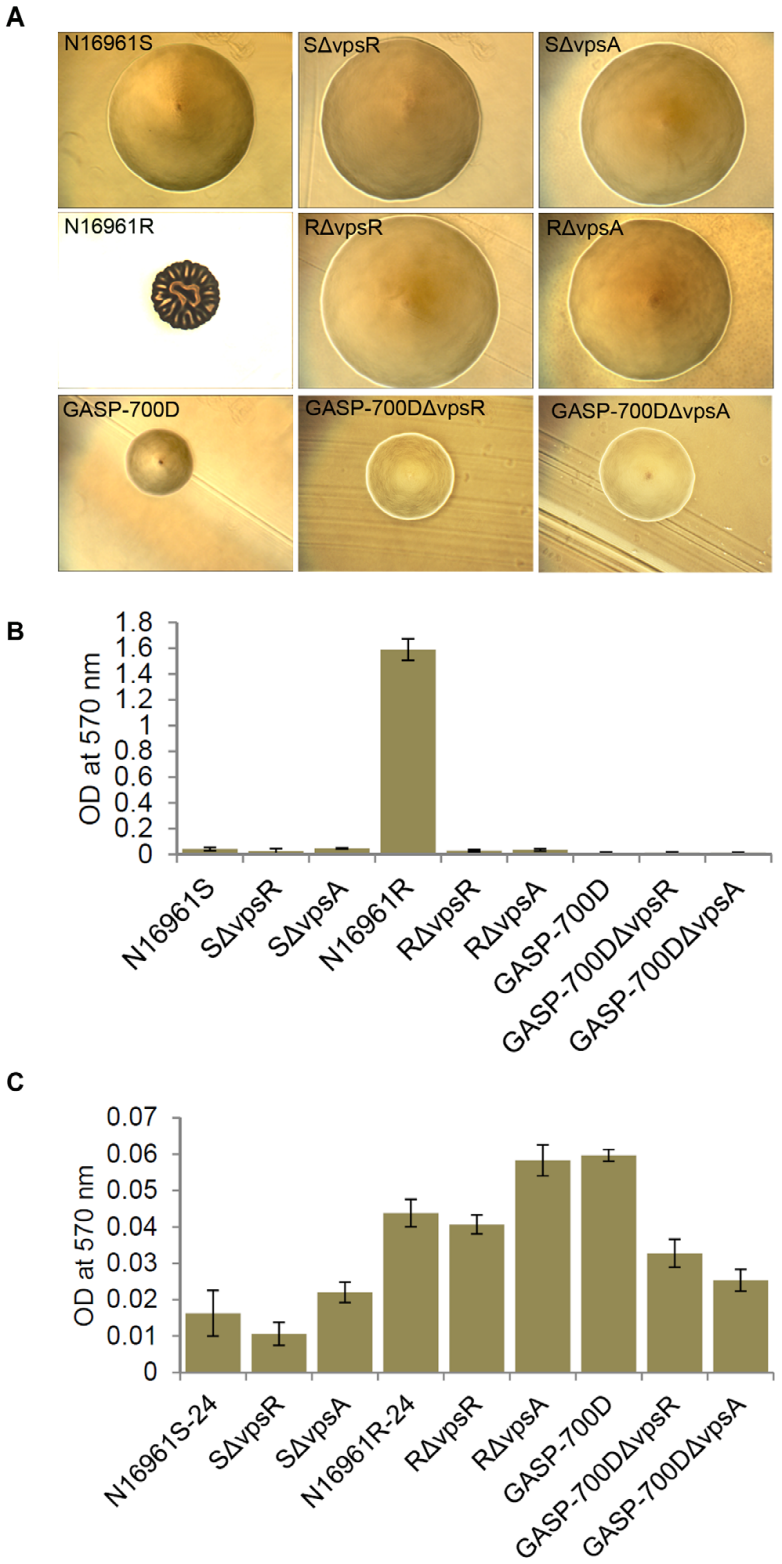
Colony morphology and associated biofilms (measured quantitatively) produced by each *V. cholerae* strain. (A) Colony morphology: each *V. cholerae* strain was subcultured on L-agar and incubated overnight at 37°C before images were acquired; (B) Quantitative measurement of biofilm produced by each *V. cholerae* strain in nutrient-rich L-broth; and (C) Quantitative measurement of biofilm produced by each *V. cholerae* strain in nutrient-poor FSLW. All the values are expressed as means ± standard deviation (SD) from at least triplicate experiments. P-values are computed by comparing the biofilm formation of each strain with that of N1961S-24 using one-way ANOVA test. A p-value of <0.005 was considered statistically significant.

We previously reported that 700 days-old persister cells showed a high degree of cell to cell aggregation compared to N16961S-24 [11]. Furthermore, the flagella of N16961S-24 allow motility, whereas GASP-700D does not facilitate productive motility. Because *V. cholerae* motility and the polar flagellum contribute to biofilm formation [19], [29], we were interested in determining the role(s) of the novel bipolar and possible non-productive/deleted peritrichous flagella elicited by N16961S-24 and GASP-700D, respectively, in biofilm formation when grown in nutrient-poor FSLW. As *V. cholerae* biofilm is produced and positively regulated by *vps* genes and *vpsR* gene, respectively, we created *vpsR* and *vpsA* in-frame deletion mutations in the background of N16961S, N16961R and GASP-700D. As expected, *vpsR* and *vpsA* mutants inhibited rugose colony phenotype (Figure 3A) [8], [24]. We initially measured biofilm production by *V. cholerae* strains, including N16961S, N16961R, GASP-700D and in-frame deletion mutants of the *vpsR* and *vpsA* biofilm genes, in the background of N16961S (SΔ*vpsR* and SΔ*vpsA*), N16961R (RΔ*vpsR* and RΔ*vpsA*) and GASP-700D (GASP-700DΔ*vpsR* and GASP-700DΔ*vpsA*) in nutrient-rich L-broth incubated overnight statically at room temperature. In L-broth, the rugose N16961R variant produced about 40- and 120-fold more biofilm (p<0.005) than its smooth counterparts N16961S and GASP-700D respectively; as expected, the N16961R mutants, RΔ*vpsR* and RΔ*vpsA* were defective for biofilm formation (Figure 3B). Our data are consistent with earlier reports that demonstrated a requirement for *vpsR* and *vpsA* for biofilm formation in nutrient-rich L-broth [22], [30].

We next examined comparative biofilm forming abilities among the *V. cholerae* strains by growing them in nutrient-poor FSLW. Interestingly, the rugose N16961R-24 variant produced 36-fold less biofilm (p<0.005) under nutrient-poor conditions (FSLW) compared to that of under nutrient-rich conditions (L-broth) (Figures 3B and 3C), demonstrating that nutrient-rich conditions favor increased *vps*-mediated biofilm formation. Interestingly, GASP-700D produced about 4-fold (p<0.005) and 1.5-fold (p = 0.24) higher biofilm compared to N16961S-24 and N16961R-24 in FSLW, respectively. In contrast to biofilm production in nutrient-rich L-broth, the Δ*vpsR* mutants (except for SΔ*vpsR*) and Δ*vpsA* mutants produced increased biofilm formation (p<0.005) in FSLW compared to N16961S-24. Our observations suggest that GASP-700D and *vpsR* and *vpsA* mutants produced biofilm in response to nutrient stress (in FSLW), and that this biofilm is somewhat independent of VPS-mediated biofilm formation as described previously [8].

**Figure 4:**
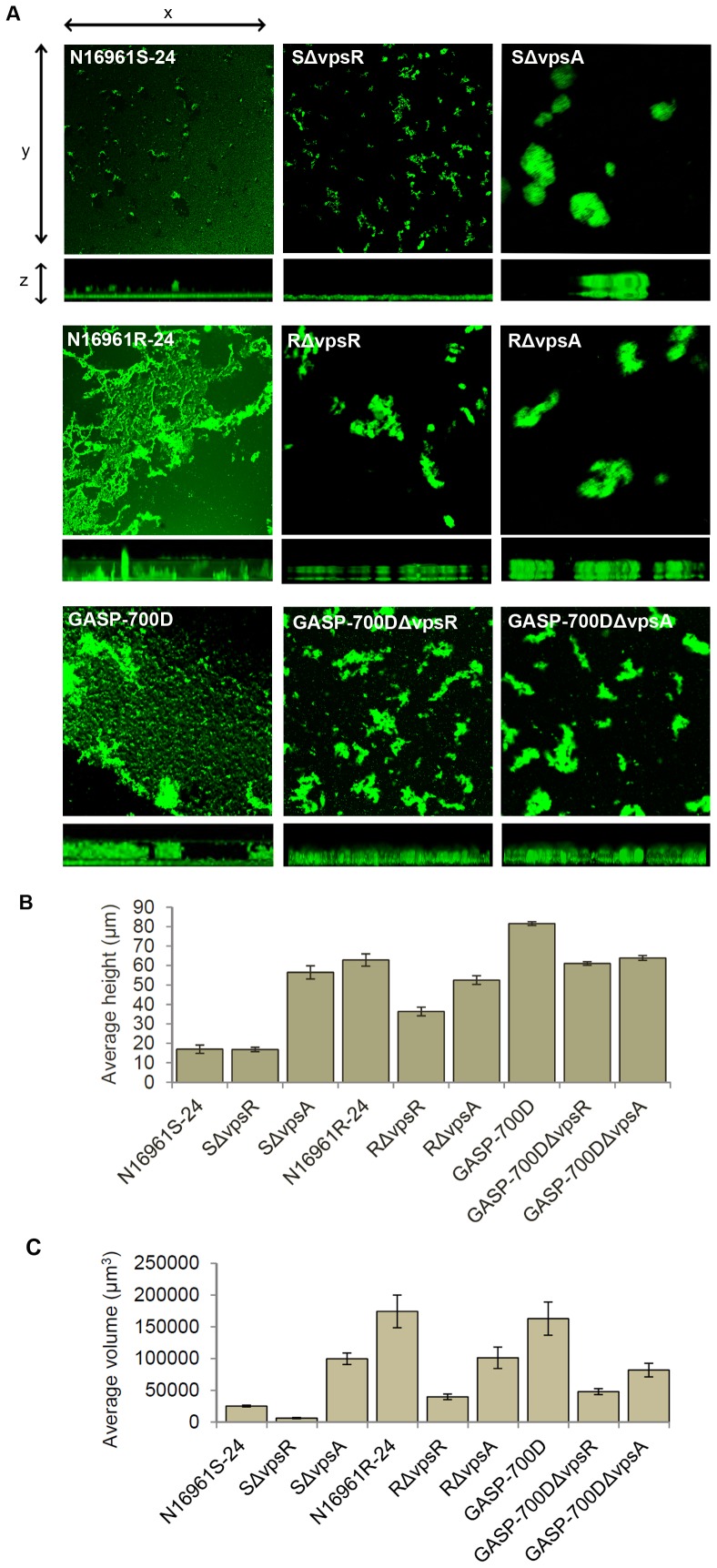
Topography and architecture of *V. cholerae* biofilms. Each strain was grown in a 4-well cell culture plate containing 500 μl FSLW. A glass cover slip was dipped into each culture well and incubated overnight statically at room temperature. The glass cover slips were stained with SYTO 9 and the images were obtained using a laser scanning confocal microscopy with an excitation and emission wavelengths of 484 and 500 nm, respectively. (A) Images of x–y sections (top panels) and x–z projections of the same biofilms (bottom panels) were analyzed with DAIME software; magnification, x200. (B) Average biofilm heights (μm) for each strain measured across five random x–z sections. (C) Total volume of biofilm (μm^3^) for each strain calculated by x–y and x–z projections. A p-value of <0.005 was considered statistically significant.

To gain further insights into the three dimensional and architectural appearances of biofilms, we examined biofilm produced by *V. cholerae* strains in FSLW described above using scanning confocal laser microscopy (SCLM). As shown in Figure 4A and 4B, the GASP-700D produced a highly-developed coalesced biofilm with 81 μm high pillars and columns filled with fluids. In contrast, the smooth variant N16961S-24 displayed a less-developed biofilm (mostly monolayer) with 17 μm pillars, and, as expected, the rugose variant produced scattered and developed biofilm with 64 μm pillar. GASP-700D appeared to be densely aggregated rather than dispersed, as in the N16961R-24 biofilms. Except SΔ*vpsR*, all Δ*vpsR* and Δ*vpsA* mutants examined exhibited patchy biofilms in FSLW with GASP-700DΔ*vpsR* and GASP-700DΔ*vpsA* displayed much higher patchy biofilms (Figure 4A). Specifically, SΔ*vpsR,* SΔ*vpsA,* RΔ*vpsR,* RΔ*vpsA,* GASP-700DΔ*vpsR* and GASP-700DΔ*vpsA* strains formed patchy biofilms with the pillars’ heights of 15, 56, 36, 52, 61 and 64 μm, respectively (Figure 4B). Figure 4C shows the quantitative analysis of biofilm formation which indicates that all strains except SΔ*vpsR* produced increased biofilm (p<0.005) compared to N16961S-24.

**Figure 5:**
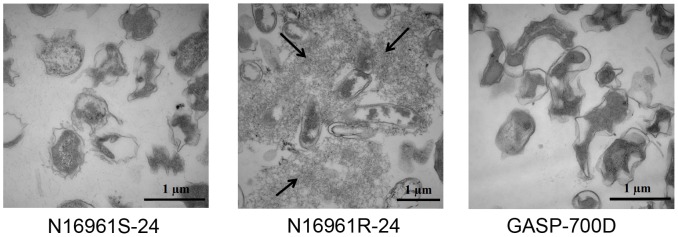
Ruthenium red staining of exopolysaccharide produced by *V. cholerae* strains. Each *V. cholerae* strain (ca. 10^8^ cfu/ml) was grown in 3 ml FSLW and incubated overnight statically at room temperature. The cultures were stained with ruthenium red stain (as described in Methods section) and images were visualized using transmission electron microscopy (TEM). Exopolysaccharide produced by N16961R-24 is indicated by arrows; N16961S-24 and GASP-700D did not develop any exopolysaccharide. Bars = 1 μm.

We stained biofilms formed by N16961S-24, N16961R-24 and GASP-700D in FSLW with ruthenium red, and examined the biofilm matrix using transmission electron microscopy (TEM) (Figure 5). Copious amounts of exopolysaccharide matrix could be detected surrounding the N16961R-24 cells, whereas very little exopolysaccharide matrix could be seen in the biofilm of N16961S-24. Likewise, GASP-700D biofilms appeared to contain very little exopolysaccharide matrix, suggesting that GASP-700D forms VPS-independent biofilms. Taken together, our data support the idea that GASP-700D produced biofilm specific to FSLW and that this biofilm is independent of VPS-mediated biofilm.

### Stress Resistance

**Figure 6:**
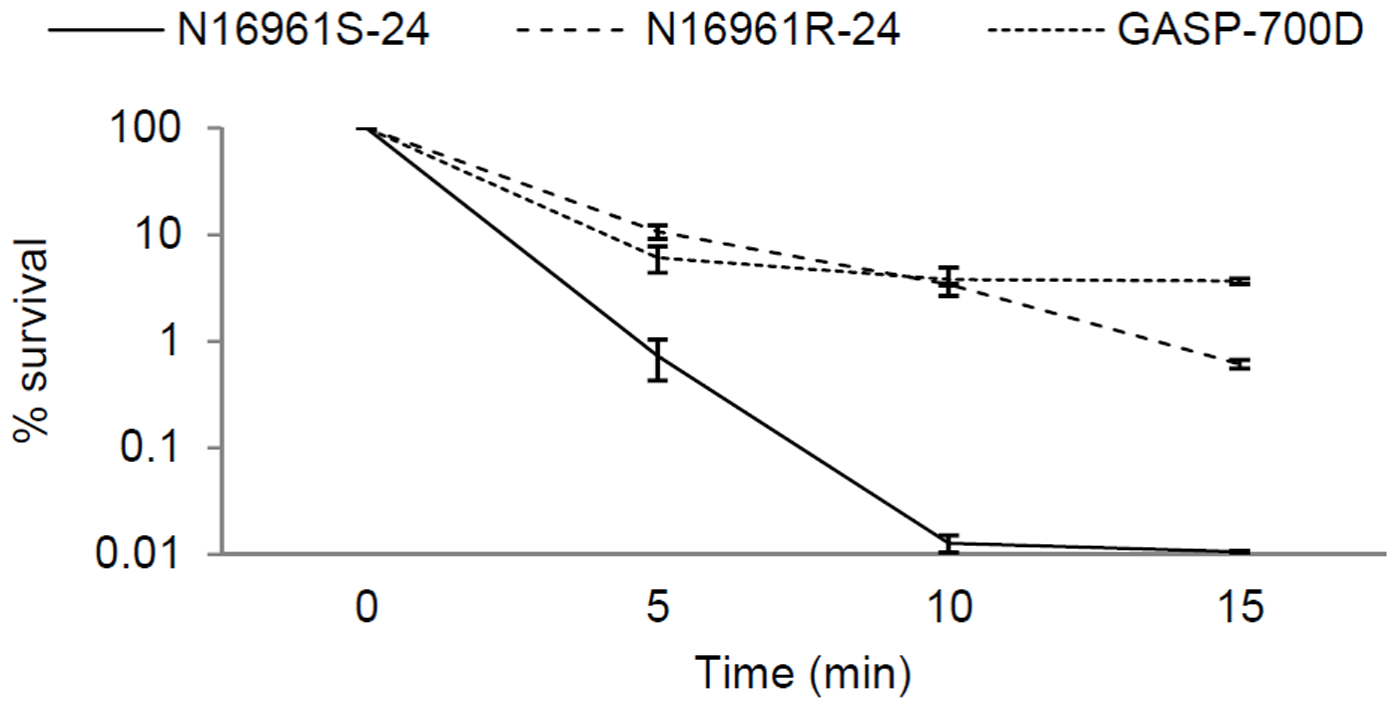
Resistance of GASP-700D to oxidative (H_2_O_2_) stress. *V. cholerae* strains N16961S-24, N16961R-24 and GASP-700D were grown (ca. 10^8^ cfu/ml) in FSLW supplemented with 20 mM H_2_O_2_. The cultures were examined at 5 min interval for 15 min for the presence of culturable bacteria as determined by standard plate count. Error bars indicate means ± standard deviation (SD) from triplicate experiments. The stress resistance of each strain was compared with that of N1961S-24 using one-way ANOVA test. A p-value of <0.005 was considered statistically significant.

We and others have previously reported that *V. cholerae* rugose variants, that produce copious amounts of exopolysaccharide and biofilm, can resist chlorine, oxidative, and osmotic stresses [8], [9], [10]. As GASP-700D produced FSLW-specific biofilm, we investigated whether this phenotype, like rugose phenotype can resist diverse stresses [31], [32]. To this context, we subjected GASP-700D to H_2_O_2,_ chlorine, and NaCl stresses. We note that there were no obvious growth differences among *V. cholerae* strains grown in L-broth and examined in this study (data not shown). Interestingly, we observed that, like N16961R-24, GASP-700D was more resistant to H_2_O_2_ in FSLW (p<0.005) compared to N16961S-24 (Figure 6). However, unlike N16961R-24, GASP-700D was as susceptible as N16961S-24 when exposed to chlorine and osmotic stresses (data not shown).

## Discussion

Recently, we reported a *V. cholerae* “persister” phenotype which is a key step in the understanding of the long-term survival of *V. cholerae* in the environment. However, substantial work still needs to be done to understand this phenotype, and to assess its role in cholera transmission. In the current study, we provide evidence that glycerol stored persister cells (700 days-old cells) have transitioned to what appeared to be a growth advantage in stationary phase (GASP) phenotype. Compared to its wild-type strains (N16961S-24 and N16961S), GASP-700D phenotype of *V. cholerae* exhibited: (i) non-motile phenotype, (ii) enhanced exopolysaccharide production and biofilm formation that are specific to FSLW, and independent of *vps*, (iii) resistance to oxidative stress, and (iv) small colony phenotype. The storage and subculture of persister cells in glycerol broth at −80°C may have influenced the observed phenotype seen with GASP-700 as described above.

We hypothesize that, during long-term survival (700 days) in stressful stationary culture, *V. cholerae* may have adopted two responses, including: (i) assume “persister” phenotype [11], and (ii) select GASP mutants that successfully adapt to stressful growth conditions [12]. Although we currently have no supporting evidence to conclude that GASP-700D genome has any mutation, we did observe that GASP-700D is defective in productive motility implying that GASP-700D may have possible mutation(s)/alteration in its genome. We propose that GASP-700D represents a GASP phenotype. Indeed, previous reports demonstrated that GASP phenotypes with genetic mutations are common in microorganisms surviving long-term in stressful and stationary growth phase.

The nutrient-poor growth conditions in FSLW affect the motility of *V. cholerae* even before its transition to GASP-700D in a phase-dependent manner. The smooth variant exhibited reduced motility in soft agar after 24 h growth in FSLW. In contrast, the rugose variant, which normally shows reduced motility in comparison with the smooth variant, was unaltered for motility after 24 h growth in FSLW. Once the bacteria have transitioned into GASP-700D, however, they appear non-motile in this assay (Figure 1). qRT-PCR revealed a dramatic downregulation (1000-fold) of *flrA* expression in GASP-700D (Figure 2). FlrA is the “master regulator” of the flagellar transcription hierarchy [33]. It is the sole Class I flagellar factor that activates σ^54^-dependent transcription of Class II flagellar genes, thus initiating flagellar synthesis. It is not known how *flrA* transcription is itself controlled in *V. cholerae*, but expression of the FlrA homologue FleQ in *Pseudomonas aeruginosa* has been shown to be negatively regulated by the alternate sigma factor AlgT, which results in loss of motility that is simultaneous with increased polysaccharide expression and biofilm formation [34]. It is not clear whether the reduction in *flrA* transcription is responsible for the non-motile phenotype, because interestingly, transcription of other flagellar genes within the transcription hierarchy, including the Class III regulator *flrC*, the motor genes *motB* and *motY*, and the major core flagellin, *flaA,* were not dramatically reduced in GASP-700D. It has been shown previously that mutation of *flhG* leads to the expression of multiple polar flagella, and the *flhG V. cholerae* strain appears non-motile in soft agar, possibly due to an inability to effectively coordinate flagellar function [35].

Previous studies of *V. cholerae* biofilm formation have mostly focused on nutrient-rich growth conditions either in static and/or in flow-cell methods [9], [36]. Under these conditions, the rugose variant produces robust biofilms with three dimensional pillars and columns [36]. Here, we studied biofilms formed in nutrient-poor FSLW conditions that more closely mimic the natural environment of *V. cholerae* [4], [37]. We found that nutrient-poor conditions promote much less biofilm formation than the nutrient-rich conditions; even with the rugose variant (Figures 3B and 3C). Our previous study demonstrated that a number of sugars, including sucrose and glucose, inhibited *V. cholerae* exopolysaccharide expression [38]. In contrast, glucose promoted biofilm production by *Staphylococcus aureus* [39], [40]. Our observations suggest that physical and chemical parameters, including nutrient composition, pH, and attachment surfaces, can influence the outcome of biofilm formation by *V. cholerae*.

GASP-700D produces a well-developed biofilm in FSLW that appears predominantly coalesced rather than scattered. In contrast, the rugose variant forms well-developed but scattered biofilms (Figure 4A). However, in the absence of the VPS genes Δ*vpsR* or Δ*vpsA*, the rugose variant forms biofilms with similar coalesced characteristics to GASP-700D in this medium, as does a SΔv*psA*, GASP-700DΔ*vpsR* and GASP-700DΔ*vpsA* mutants (Figures 4A and 4B). This suggests that GASP-700D and the strains lacking *vps* genes form biofilms that are independent of VPS, and that *vps* genes may negatively affect the expression of the alternative biofilm matrix. Ruthenium red staining failed to detect exopolysaccharide in the GASP-700D biofilms in FSLW (Figure 6), in contrast to the abundant exopolysaccharide in the rugose variant biofilms, suggesting that the GASP-700D biofilms may contain yet to be defined biofilm matrix. Such a putative extracellular matrix could drive the development of the alternative, coalescing biofilms seen in the GASP-700D which is more resistant to oxidative stress than either smooth or rugose variants. Oxidative stress resistance may be due to the alternative biofilms formed under these conditions, or alternatively, to enhanced expression of resistance factors. We are currently performing further genetic investigations of GASP-700D in order to enhance our understanding if *V. cholerae* GASP-700D sustained mutation(s) to select GASP mutants as seen with other microorganisms [12] and thereby promoting competitive environmental fitness and adaptation.

## Supporting Information

Figure S1 **Comparative analysis of the differential gene expression among** ***V. cholerae*** **strains N16961S and GASP-700D using qRT-PCR. N16961S was grown both in nutrient-rich L-broth and in nutrient-poor FSLW (N16961S-24) (ca. 10^8^ cfu/ml), and the cultures were incubated overnight statically at room temperature.** GASP-700D was grown (ca. 10^8^ cfu/ml) in FSLW only. Expression of each gene was normalized to that of *toxR*, and subsequently compared to that of the wild-type N16961S grown in L-broth. Data represent the average results of three independent experiments and error bars indicate as means ± standard deviation (SD).
(TIF)

Table S1 **Oligonucleotide primers used in this study.**
(DOCX)
