# Supplementary figures and images for: Vibrio cholerae Persisted in Microcosm for 700 Days Inhibits Motility but Promotes Biofilm Formation in Nutrient-Poor Lake Water Microcosms
Source: PLoS One. 2014 Mar 25;9(3):e92883. doi: 10.1371/journal.pone.0092883 (PMC3965490; doi:10.1371/journal.pone.0092883)

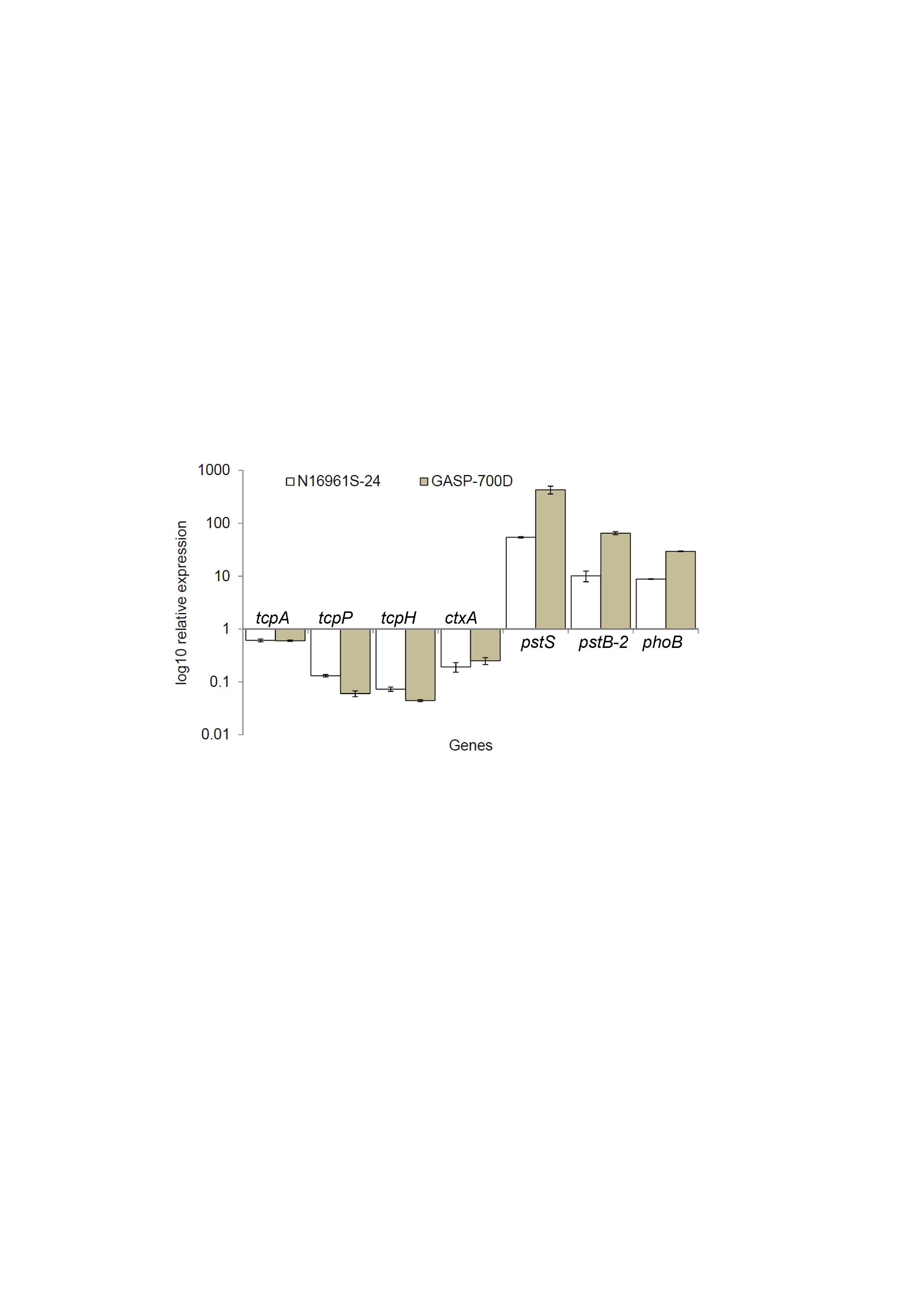

Supplement: Figure S1 — Comparative analysis of the differential gene expression among V. cholerae strains N16961S and GASP-700D using qRT-PCR. N16961S was grown both in nutrient-rich L-broth and in nutrient-poor FSLW (N16961S-24) (ca. 108 cfu/ml), and the cultures were incubated overnight statically at room temperature. GASP-700D was grown (ca. 108 cfu/ml) in FSLW only. Expression of each gene was normalized to that of toxR, and subsequently compared to that of the wild-type N16961S grown in L-broth. Data represent the average results of three independent experiments and error bars indicate as means ± standard deviation (SD). (TIF) [file pone.0092883.s001.tif]
