# Supplementary material for: Vibrio cholerae Persisted in Microcosm for 700 Days Inhibits Motility but Promotes Biofilm Formation in Nutrient-Poor Lake Water Microcosms
Source: PLoS One. 2014 Mar 25;9(3):e92883. doi: 10.1371/journal.pone.0092883 (PMC3965490; doi:10.1371/journal.pone.0092883)
Supplement: Table S1 — Oligonucleotide primers used in this study. (DOCX) [file pone.0092883.s002.docx]

Table S1. Oligonucleotide primers used in this study

| Primer types | Primer name | Direction | Target Gene | Sequence (5’ to 3’)^a^ |
| --- | --- | --- | --- | --- |
| PCR | |  |  |  |
|  | aa212 | Forward | *vpsR* | TCC CCG CGG ATG AGC ACT CAA TTC CG |
|  | aa213 | Reverse | (1^st^ fragment) | GCT CTA GAC GAG CCA AAA TGT GGC C |
|  | aa214 | Forward | *vpsR* | GCT CTA GAT CAG ACA TCG AAA AAG C |
|  | aa215 | Reverse | (2^nd^ fragment) | CGG GAT CCT TAG AAG TTT TCA TCG G |
|  | aa264 | Forward | *vpsA* | TCC CCG CGG GCA AGG CGA ATC GAC AAG |
|  | aa265 | Reverse | (1^st^ fragment) | GGA CTA GTC ACT TCC CCA CAT CCT CT |
|  | aa266 | Forward | *vpsA* | GGA CTA GTG CGA AAT AGA CTC ATC AGG GG |
|  | aa267 | Reverse | (2^nd^ fragment) | GGA ATT CGC CAT TGC GCC AAT TTT TCG G |
| Real time PCR | |  |  |  |
|  | aa125 | Forward | *flaA* | TAA CAG TGC AAA AGA TGA CG |
|  | aa126 | Reverse |  | GTT TGA GCA ATC GAA ATA CC |
|  | aa127 | Forward | *flrC* | AAG CAA AGT CTT AAT CGT AG |
|  | aa128 | Reverse |  | ACA CTG TGT GAT TTG AGT TT |
|  | aa133 | Forward | *phoB* | ATG TCT AGA AGG ATT CTG GT |
|  | aa134 | Reverse |  | GAA TCA TAA TCT TCA GCC TC |
|  | aa135 | Forward | *pstB2* | TGA ATC GTA TGA ATG ATC TC |
|  | aa136 | Reverse |  | TCA TAA ATA CTC ATT GGG AA |
|  | aa137 | Forward | *pstS* | CAC TTA TAG GAA ATT CGT GA |
|  | aa138 | Reverse |  | ACT GCT GAG ATT GTC TCT TT |
|  | aa139 | Forward | *tcpA* | CAA ACT TAT CGT AGT CTT GG |
|  | aa140 | Reverse |  | CAT AGC TGT ACC AGT GAA AG |
|  | aa141 | Forward | *tcpP* | GAA TGA ATG CAC TAA TCA AG |
|  | aa142 | Reverse |  | ATT ATT TGA TCA TTT GGA CA |
|  | aa143 | Forward | *toxR* | ATA TCG ATG AGT CAT ATT GG |
|  | aa144 | Reverse |  | TAA TCG AAT GAT CTC TTC AC |
|  | aa180 | Forward | *tcpH* | GTG TAA CGA TCA TCG CAC TC |
|  | aa181 | Reverse |  | ATA GGT TAC AAA CCG AAT GG |
|  | aa254 | Forward | *motB* | GAA CAA CAA TGT AAA TGT CC |
|  | aa255 | Reverse |  | TAA ATT TCA GTA CGT CCA TC |
|  | aa256 | Forward | *motY* | CCC TAC AAT GAG TTT AGT GA |
|  | aa257 | Reverse |  | GCT TTA TTC AAC TCA ACA CT |
|  | aa258 | Forward | *flrA* | GAG TTT AGC GAA ACT ACT TG |
|  | aa259 | Reverse |  | TAG ACT CAA TGA CCT CAC AC |
|  | aa260 | Forward | *ctxA* | TCT AGA CCT CCT GAT GAA AT |
|  | aa261 | Reverse |  | AAG GTT GAT ATT CAT TTG AG |

^a^Underline sequences represent restriction sites
